# Short-term association between ambient temperature and homicide in South Africa: a case-crossover study

**DOI:** 10.1186/s12940-019-0549-4

**Published:** 2019-12-16

**Authors:** Abigail Gates, Mitchel Klein, Fiorella Acquaotta, Rebecca M. Garland, Noah Scovronick

**Affiliations:** 1 Department of Environmental Health, Rollins School of Public Health, Emory University, Atlanta, GA USA; 2 Department of Earth Sciences, University of Turin, Turin, Italy; 3 Smart Places Cluster, Council for Scientific and Industrial Research, Pretoria, South Africa; 4 Department of Geography, Geoinformatics and Meteorology, University of Pretoria, Hatfield, Pretoria, South Africa; 5 Unit for Environmental Sciences and Management, North West University, Potchefstroom, South Africa

**Keywords:** Temperature, Weather, Climate, Crime, Homicide, South Africa

## Abstract

**Background:**

Criminology research has traditionally investigated sociodemographic predictors of crime, such as sex, race, age, and socioeconomic status. However, evidence suggests that short-term fluctuations in crime often vary more than long-term trends, which sociodemographic factors cannot explain. This has redirected researchers to explore how environmental factors, such as meteorological variables, influence criminal behavior. In this study we investigate the association between daily ambient temperature and homicide incidence in South Africa, a country with one of the highest homicide rates in the world.

**Methods:**

Mortality data was from South Africa’s civil registration system and includes all recorded deaths in the country from 1997 to 2013 (17 years). Daily temperature was from the National Oceanographic and Atmospheric Association of the United States and South Africa’s Agricultural Research Council. Data were analyzed using a time-stratified case-crossover design with conditional logistic regression. We delineated cases as either “definite” (ICD-10 codes X85-Y09, *n* = 68,356) or “probable” homicides (ICD-10 codes W25-W26, W32-W34, W50, Y22-Y24, Y28-Y29, *n* = 177,873). Case periods were defined as the day on which a death occurred. Control periods were selected using a day-of-week match within the same month and district. Analyses investigated same-day and lagged effects of maximum, mean and minimum temperature.

**Results:**

A one-degree Celsius increase in same-day maximum temperature – our a priori metric of choice – was associated with a 1.5% (1.3–1.8%) increase in definite homicides and a 1.2% (1.1–1.3%) increase in total (definite + probable) homicides. Significant (*p* < 0.05) positive associations were also observed when applying other temperature metrics (mean, minimum) and lags (1, 0–1). The shape of the association did not display any clear non-linearities. There was no evidence of confounding by public holidays or air pollution.

**Conclusions:**

This study suggests a positive association between daily ambient temperature and homicide in South Africa. This temperature-health relationship may be of particular concern in the context of climate change. The ability to include meteorological variables as a predictor of criminal activity and violent behavior could prove valuable in resource allocation for crime prevention efforts.

## Background

Criminology has traditionally investigated sociodemographic predictors of crime, such as sex, race, age, and socioeconomic status. However, evidence suggests that short-term fluctuations in crime, which sociodemographic factors typically cannot explain, often vary more than long-term trends [1]. This has redirected researchers to explore how environmental factors, such as meteorological variables, may also influence criminal behavior. Several theories have been proposed to explain possible mechanisms underlying these relationships, including that temperature may influence behavior, for example by changing the probability of convergence between a likely offender and suitable target, or by altering patterns of alcohol consumption (both consistent with Routine Activity Theory) [2, 3]. Biological theories suggest that weather changes or extremes may act as a stressor that facilitates aggression [4–7].

Previous studies indicate that ‘violent crime’ as a broad category (often including homicide, assault, burglary, domestic violence and/or rape) may be positively associated with ambient temperature [1, 4, 5, 8–21], but evidence on the relationship between temperature and homicide specifically is more limited [3, 11, 13–17, 22, 23]. A recent paper [24] on the links between heat and homicide reviewed 16 studies, noting that nine reported a significant positive association whereas the others reported positive but non-significant associations. The paper also raised concerns about the relationship between climate change and violence in South Africa specifically.

The aim of this study is to investigate the short-term association between daily ambient temperature and homicide in South Africa using a national dataset that includes all recorded deaths in the country from 1997 to 2013. South Africa has one of the highest homicide rates on record at an estimated 36 per 100,000 individuals, roughly six times the global average [25]. South Africa is also experiencing climate warming at a faster rate than the global average [26, 27], which heightens the importance of understanding potential relationships between temperature and adverse health and social outcomes such as homicide.

## Materials and methods

### Mortality data

Mortality data was derived from a dataset of all recorded deaths in South Africa from 1997 to 2013 (17 years) provided by Statistics South Africa, the country’s national statistical service. Reported deaths include information on the date of death, cause of death according to the 10th revision of the International Classification of Disease (ICD), and the location of death at the level of district municipality, of which there are 52 in South Africa (hereafter referred to as “districts”).

As violent deaths are known to be under/misreported in South Africa [28, 29], we define two categories of cases. The first category includes cases where the immediate or underlying cause of death was recorded as a homicide (ICD-10 codes X85-Y09). We refer to this category as “definite” homicides. The second category includes violent deaths recorded as accidental or of undetermined intent that we consider likely to be homicides based on prior literature exploring this issue (ICD-10 codes W25-W26, W32-W34, W50, Y22-Y24, Y28-Y29) [28–30]; we refer to this group as “probable” homicides. The two groups together comprise the “total” homicides. Cause of death reporting is discussed in more detail in the Discussion.

### Temperature data

Daily temperature was derived from a dataset prepared for a prior study investigating temperature-mortality relationships in South Africa [31], and is based on data from the National Oceanographic and Atmospheric Association of the United States and South Africa’s Agricultural Research Council. The dataset underwent a quality control procedure to remove illogical measurements, outliers and duplicate sets (periods with at least five consecutive days recording the same temperature). Next, the series were tested for homogeneity and corrected if necessary for breaks in recording or changes/relocation of instruments [32]. After the quality control procedure, we assembled a final composite dataset consisting of one station for each district (see Additional file 1: Figure S1 for a map of South Africa and the location of the stations). The preferred station in each district was selected using the CoTemp software and based on the length of the series, the start and end dates of the measurements, and accounting for gaps and homogeneity in the series [33–35]. Missing data were reconstructed using nearby comparison stations for data gaps less than or equal to 6 days in length; in total, we reconstructed 7% of daily maximum values and 12% of daily minimum values [31]. There were temperature data available for 29 districts at the beginning of the study period, increasing to cover all 52 districts by 2013 (Additional file 1: Figure S2) [31].

### Statistical approach

Conditional logistic regression models were fit using SAS version 9.4 (SAS Institute Inc., Cary, North Carolina), with results reported as odds ratios per °C increase in temperature. We applied a bidirectional time-stratified case-crossover approach in which a case period was defined as the day on which a death by homicide occurred, with control periods matched on day of the week, month and district. In other words, each case acts as its own control. By design, this approach controls for day of the week, individual-level factors that do not change over the course of a month, and long-term trends, and typically yields 3–4 control periods per case. We pooled all deaths into a single national dataset, but tested for interaction by district through the inclusion of product terms between temperature and district.

Our a priori preferred temperature metric was same-day (lag 0) maximum temperature, as the maximum temperature series had the fewest missing observations and is less vulnerable to improper instrument management [31, 36–41]; nevertheless, we also report results for other temperature metrics (mean and minimum) and lags (lag 1, the previous day’s temperature, and a lag 0–1 moving average). The estimation of the temperature effect on homicide is derived by contrasting case days with control days, which are within-month, same day of week comparisons. Thus the estimated temperature effects are short-term effects and not the effects of seasonal changes in temperature. The temperature data were included in the models as a continuous variable and study periods with missing temperature data were excluded from analysis. In addition, to explore potential non-linearities in the effect, we estimated the temperature-homicide relationship for different intervals of same-day maximum temperature using indicator variables for temperatures between 20 and 24.9 °C, 25–29.9 °C, 30–34.9 °C, and 35+ °C; temperatures below 20 °C served as the reference group, which was chosen to be large enough to have sufficient precision, but also at a logical cutpoint to allow for easy comparison with other studies.

We analyzed the data over the whole study period, but to control for the possibility of higher levels of underreporting in the early years of the study period we also performed a restricted analysis to include only 2002–2013, thus omitting the first 5 years of data. In other sensitivity analyses we ran models separately after excluding holidays and controlling for air pollution (PM_2.5_ and ozone, lag 0) in those districts for which we had data (*n* = 6 districts for PM_2.5_ and *n* = 5 districts for ozone). As femicide from intimate-partner violence is of high concern in South Africa [42, 43], we also report results separately for male and female deaths, as well as by age of the victim for ages 0–17, 18–44, and 45+. We did not have information on socioeconomic status.

## Results

There were 246,229 cases across all districts over the full study period, including both definite (*n* = 68,356) and probable (*n* = 177,873) homicides, which equates to a mean of ~ 40 per day (Table 1). Maximum daily temperature ranged from 0.5 °C to 47.8 °C (Table 1).

**Table 1:** Descriptive statistics for temperature and mortality data. Temperature data is for the case/control periods only

	Mean	Max	75%	Median	25%	Min	Missing temperature data (%)	
							Case days	Control days
Daily Temperature (°C)								
Max	24.7 ± 5.4	47.8	28.4	25.1	21.1	0.5	10.1	10.0
Mean^a^	18.0 ± 5.0	34.7	21.8	18.5	14.4	−1.1	11.5	11.4
Min	11.4 ± 5.8	28.0	15.6	12.0	7.5	−13.8	11.1	11.0
Daily Homicides^b^								
	39.7 ± 23.1	188	50	33	24	2	–	–

^a^Here the mean is defined as the average between the maximum and the minimum

^b^Homicide case counts include definite homicides (ICD-10: X85-Y09) and probable homicides (ICD-10: W25-W26, W32-W34, W50, Y22-Y24, Y28-Y29)

A one-degree Celsius increase in same-day maximum temperature – our a priori preferred temperature metric – was associated with a 1.5% (1.3–1.8%) increase in definite homicides and a 1.2% (1.1–1.3%) increase in total (definite + probable) homicides (Table 2). There was some evidence of heterogeneity by district (I^2^ = 35.1% for definite and I^2^ = 21.2% for total homicides) and therefore in Additional file 1: Table S1 we report district-specific results for all 52 districts and also aggregate those district-level estimates in a random-effects meta-analysis; this sensitivity test produced very similar country-level associations. Results by province are reported in Additional file 1: Table S2. Results did not meaningfully change when including air pollution, when restricting analyses to later years or when excluding holidays (Additional file 1: Table S3).

**Table 2:** Association between daily temperature and homicide, with odds ratios reported per 1 °C increase in temperature. Numbers in parentheses are 95% confidence intervals

	Deaths included		Odds ratio	
	Definite^a^	Total^b^	Definite^a^	Total^b^
Max				
Lag 0^c^	63,343	223,733	1.015 (1.013, 1.018)	1.012 (1.011, 1.013)
Lag 1^d^	63,335	223,735	1.012 (1.009, 1.014)	1.008 (1.007, 1.010)
Lag 0-1^e^	63,131	223,035	1.018 (1.015, 1.021)	1.014 (1.012, 1.015)
Mean^f^				
Lag 0^c^	62,601	220,854	1.021 (1.017, 1.024)	1.017 (1.015, 1.019)
Lag 1^d^	62,661	220,999	1.016 (1.012, 1.019)	1.011 (1.009, 1.013)
Lag 0-1^e^	62,200	219,234	1.023 (1.019, 1.027)	1.018 (1.015, 1.020)
Min				
Lag 0^c^	62,850	221,724	1.010 (1.007, 1.013)	1.009 (1.007, 1.011)
Lag 1^d^	62,890	221,874	1.007 (1.004, 1.010)	1.005 (1.004, 1.007)
Lag 0-1^e^	62,502	220,320	1.011 (1.008, 1.015)	1.010 (1.008, 1.012)

^a^Definite homicides, ICD-10: X85-Y09

^b^Includes definite homicides and probable homicides (ICD-10: W25-W26, W32-W34, W50, Y22-Y24, Y28-Y29)

^c^Same day’s temperature

^d^Previous day’s temperature

^e^Moving average of same and previous day’s temperature

^f^Here the mean is defined as the average between the maximum and the minimum

We also found significant positive associations between ambient temperature and odds of death by homicide for all the other temperature metrics and lags investigated (Table 2); the strongest effects were generally observed for mean temperature and lag 0–1, whereas relatively lower effects were observed for minimum temperature and lag 1.

The central estimates of the effect of maximum temperature were generally slightly elevated in male compared to female victims, although the differences were non-significant, as shown by the overlapping 95% confidence intervals (Table 3). There was also no clear evidence of differences across age groups. Results by sex and age for mean and minimum temperature are reported in Additional file 1: Tables S4 and S5.

**Table 3:** Association by age and sex between daily maximum temperature and homicide, with odds ratios reported per 1 °C increase in temperature. Numbers in parentheses are 95% confidence intervals

	Deaths included		Odds ratio	
	Definite^a^	Total^b^	Definite^a^	Total^b^
Male				
Lag 0^c^	54,278	188,737	1.016 (1.013, 1.018)	1.012 (1.011, 1.014)
Lag 1^d^	54,266	188,713	1.012 (1.009, 1.014)	1.009 (1.007, 1.010)
Lag 0-1^e^	54,090	188,141	1.018 (1.015, 1.021)	1.014 (1.012, 1.016)
Female				
Lag 0^c^	8805	34,053	1.013 (1.006, 1.019)	1.010 (1.007, 1.014)
Lag 1^d^	8809	34,081	1.013 (1.007, 1.020)	1.008 (1.004, 1.011)
Lag 0-1^e^	8781	33,953	1.017 (1.010, 1.025)	1.012 (1.008, 1.016)
0–17 years				
Lag 0^c^	4111	14,316	1.014 (1.005, 1.024)	1.011 (1.006, 1.016)
Lag 1^d^	4115	14,328	1.013 (1.004, 1.022)	1.010 (1.005, 1.015)
Lag 0-1^e^	4096	14,275	1.018 (1.008, 1.029)	1.014 (1.008, 1.020)
18–44 years				
Lag 0^c^	48,201	162,578	1.015 (1.012, 1.018)	1.013 (1.011, 1.014)
Lag 1^d^	48,200	162,560	1.011 (1.008, 1.013)	1.008 (1.006, 1.010)
Lag 0-1^e^	48,053	162,072	1.017 (1.014, 1.020)	1.014 (1.012, 1.015)
45+ years				
Lag 0^c^	10,425	44,803	1.016 (1.010, 1.022)	1.011 (1.008, 1.014)
Lag 1^d^	10,415	44,811	1.016 (1.010, 1.022)	1.010 (1.007, 1.013)
Lag 0-1^e^	10,378	44,657	1.022 (1.015, 1.029)	1.014 (1.010, 1.017)

^a^Definite homicides, ICD-10: X85-Y09

^b^Includes definite homicides and probable homicides (ICD-10: W25-W26, W32-W34, W50, Y22-Y24, Y28-Y29)

^c^Same day’s temperature

^d^Previous day’s temperature

^e^Moving average of same and previous day’s temperature

**Fig. 1:**
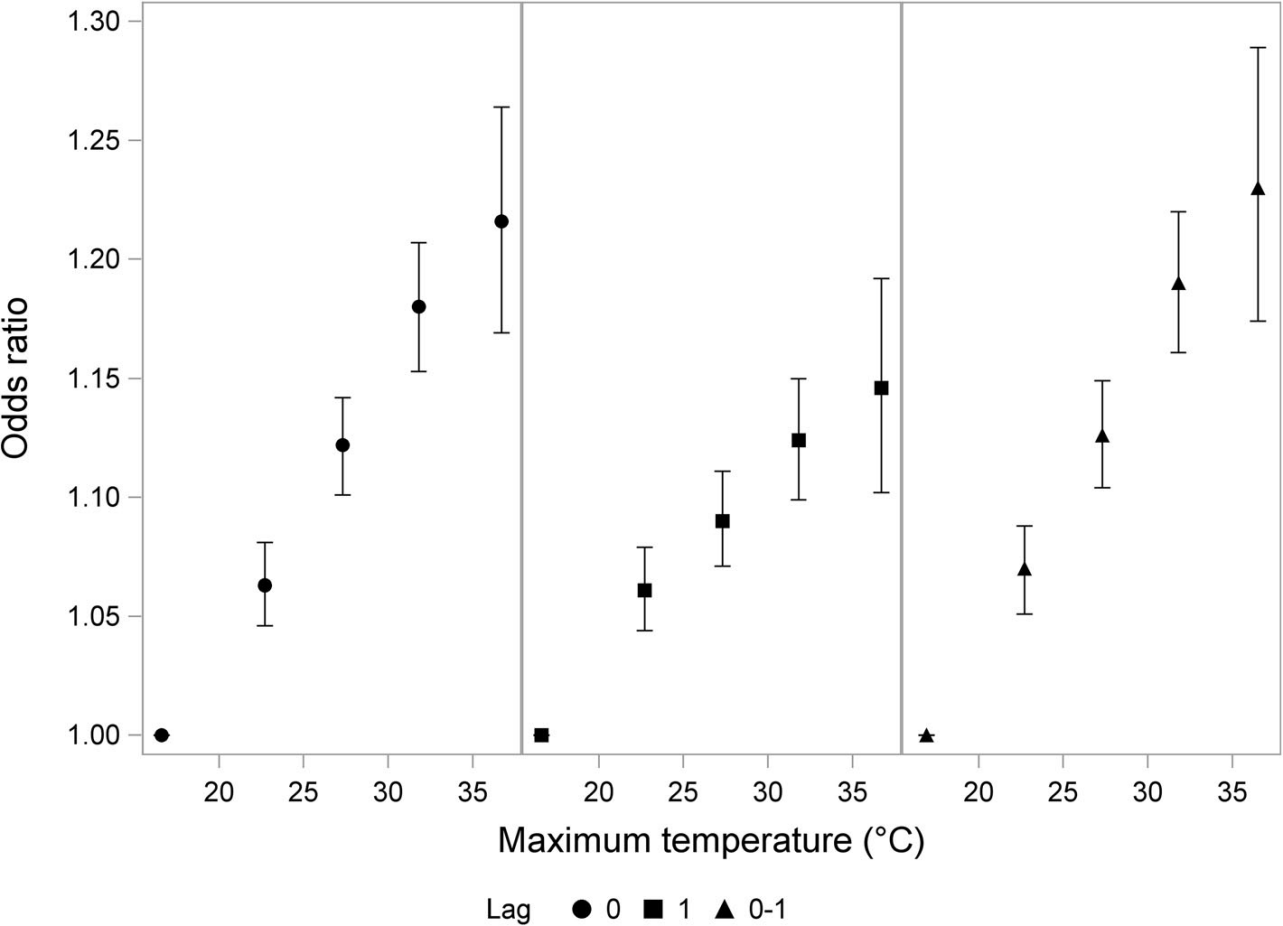
Association between maximum temperature and homicide using intervals of temperature in comparison to the reference group of <20 °C. Odds ratios are plotted at the mean temperature within each 5 °C interval. Bars indicate 95% confidence intervals

Figure 1 displays results when grouping the cases into temperature intervals, thus enabling an exploration of the shape of the association. The temperature-homicide association did not exhibit any clear non-linearities, although results in the highest temperature group (35 °C+) were more uncertain – due to the relatively low number of days in that group – and thus do not rule out the possibility of a diminishing marginal effect on extremely hot days. According to this categorical approach, the odds of homicide when same-day maximum temperatures are between 30 °C and 35 °C were an estimated 18% higher than when temperatures are below 20 °C (95% CI 1.155–1.206).

## Discussion

The South African context offers a unique opportunity to explore the temperature-homicide association due to the country’s high homicide rate combined with our long-term dataset. Our findings indicate evidence of a positive association between short-term increases in ambient temperature and the odds of death by homicide. Our a priori preferred temperature metric was same-day maximum temperature, for which we observed an odds ratio of 1.015 (1.013, 1.018) for “definite” homicides and 1.012 (1.011, 1.013) for total (“definite” + “probable”) homicides. The associations did not display any clear non-linearities. Although the associations are modest, they remained significant when using any of the other temperature metrics and lags; the highest associations were for mean temperature and lag 0–1. To put the 1.015 effect size into context, and based on 2017/2018 homicide data from the South African Police Service (SAPS) reporting an average of ~ 56 deaths per day, we estimate that a 35 °C day would see roughly 11 more homicides on average compared to a 22 °C day.

Overall, our findings align with the existing literature investigating temperature and broad categories of violent crime. For example, a recent study in the United States investigated 301 counties across 34 states, and reported a significant and approximately linear positive association between violent crime and daily temperature [21]. A study in Baltimore, Maryland reported an association between maximum temperature and increased rates of total and violent crime [16]. Similarly, a study in Cleveland, Ohio reported a linear association between apparent temperature and aggressive crime, which included homicide [9]. In Philadelphia, Pennsylvania, researchers conducted a time series analysis of daily temperature and crime and reported that violent crime is highest when temperatures are “comfortable” [20]. There are two South African studies conducted in Tshwane (Pretoria) on weather/climate and violent crime; one reported a 50% increase in violent crime on hot days compared to very cold days [44], while the other found that less affluent neighborhoods had higher rates of assault in the summer than more affluent neighborhoods, but similar rates during the winter [45].

A strength of our study is that it is one of the few to distinguish homicide from other types of violent crime, as the existing research on the topic is more limited. Researchers in Dallas, Texas reported a very small (<0.1%) increase in the daily homicide rate for each °F increase through 89 °F, which reversed at higher temperatures [11]. Researchers examining numerous environmental factors in relation to daily crime in four U.S. cities (Chicago, Illinois; Houston, Texas; Philadelphia, Pennsylvania; and Seattle, Washington) reported a large positive but non-significant association between apparent temperature and homicide between the 75th percentile temperature compared to the 25th (risk ratio = 1.85 (0.47,7.31)) [14]. In Mashhad, Iran, researchers did not find an association between homicide and temperature or any other meteorological variable [23]. In South Africa, a study based on SAPS and weather data with monthly resolution from 2001 to 2013 reported a significant effect (coefficient = 0.015) suggesting higher levels of homicide during higher temperatures [46].

This study has several limitations. Perhaps the most important pertains to potential underreporting and/or misclassification of deaths by homicide. Although Statistics South Africa estimates that the national registry captures ~ 90% or more of all deaths during the study period [31, 47, 48], South African law discourages pathologists from reporting the manner of violent death in order to avoid prejudicing a potential investigation [29]. As a result of this and other factors, many homicides are likely to be recorded as having a different ICD-code, probably as a violent death of unintentional intent or as an accident [29]. We attempted to circumvent this issue by also including these “probable” homicides, which made the total number of cases in our study roughly consistent with the levels estimated by other researchers [28, 29, 49] and the SAPS; specifically, we were able to compare our data with SAPS data over the final five fiscal years of our study period and our total homicide number was ~ 90% of what was reported by the SAPS [25]. The slightly lower effect estimates observed in the total compared to the “definite” homicides is not surprising, as the latter category does presumably include some non-homicides, for example true accidents and/or suicides, with the latter known to (also) be misreported in South Africa [29, 50]. In general, we would expect any bias due to misclassification to be in the direction of the null as we would not expect the underreporting of homicides to be related to short term fluctuations of temperaure. In this scenario, such misclassification should not systematically lead to an estimated spurious effect.

The temperature dataset posed additional challenges. Missing temperature data resulted in the exclusion of 10.1–11.5% of cases, depending on which temperature metric was applied. Furthermore, the dataset assigned one temperature for all deaths within a district, which may not adequately capture temperature heterogeneity across larger districts in particular. This could lead to possible exposure misclassification, although we expect this issue is minimized by the fact that total homicide mortality was concentrated in the smaller districts; 51% of total cases occurred in the ten (19%) smallest districts.

We performed stratified analyses by age and sex, as prior research suggests that these characteristics may affect the type of interpersonal conflict leading to homicide, the weapon used, and/or the location of the crime, amongst other factors [51, 52]. We did not find any clear evidence of interactions by either variable, but because our dataset did not include any specifics about the circumstances of the homicides (e.g. information about the perpetrator), we encourage future research on these topics. Additional areas for future research include the exploration of other meteorological variables (e.g., humidity or temperature variability), the use of other types of crime datasets such as police service or surveillance data, and where possible, additional subgroup analysis such as by socioeconomic status.

The temperature-homicide relationship may be of particular concern in the context of climate change, especially in the absence of short-term adaptation. Identification of areas and populations at risk for higher levels of violence due to short-term changes in temperature, combined with a more thorough understanding of the pathways by which these changes influence behavior, could prove beneficial to policy measures such as those employed during heatwaves [24]. This ability to include meteorological variables as a predictor of criminal activity and violent behavior could prove valuable in resource allocation for violence prevention efforts and preparedness for first responders and healthcare providers.

## Supplementary information

**Additional file 1 **Supplementary material including additional tables and figures. **Figure S1.** Provincial and district boundaries of South Africa. **Figure S2.** Number of districts with temperature data. **Table S1** Associations between same-day maximum temperature and homicide by district. **Table S2** Associations between same-day maximum temperature and homicide by province. **Table S3** Sensitivity analyses. **Table S4** Association by age and sex between daily mean temperature and homicide. **Table S5** Association by age and sex between daily minimum temperature and homicide.

## Data Availability

The mortality data that underlies the findings of this study were supplied by Statistics South Africa. The temperature data used in this study were from the National Oceanographic and Atmospheric Association of the United States and South Africa’s Agricultural Research Council. The air quality data were supplied to the team from the South African Air Quality Information System managed by the South African Weather Service (SAWS) and from Eskom.

## Abbreviations

ARC: Agricultural Research Council (South Africa)
ICD: International Classificiation of Disease
NOAA: National Oceanographic and Atmospheric Administration
PM_2.5_: Particulate matter 2.5
SAPS: South African Police Service
