# Supplementary material for: Short-term association between ambient temperature and homicide in South Africa: a case-crossover study
Source: Environ Health. 2019 Dec 16;18:109. doi: 10.1186/s12940-019-0549-4 (PMC6915878; doi:10.1186/s12940-019-0549-4)
Supplement: Supplementary file 1 — Additional file 1 Supplementary material including additional tables and figures. Figure S1. Provincial and district boundaries of South Africa. Figure S2. Number of districts with temperature data. Table S1 Associations between same-day maximum temperature and homicide by district. Table S2 Associations between same-day maximum temperature and homicide by province. Table S3 Sensitivity analyses. Table S4 Association by age and sex between daily mean temperature and homicide. Table S5 Association by age and sex between daily minimum temperature and homicide. [file 12940_2019_549_MOESM1_ESM.pdf]

Supplementary Material

Short-term association between ambient temperature and homicide in South Africa: a case-crossover study

Abigail Gates, Mitchel Klein, Fiorella Acquaotta, Rebecca M Garland, Noah Scovronick

Figures

Figure S1 Provincial and district boundaries of South Africa, location of monitoring temperature stations and source of data 1

Figure S2 Number of districts with temperature data over the study period 2

Tables

Table S1 Associations between same-day maximum temperature and homicide by district, tests for interaction and heterogeneity, and overall country-level association estimated by random-effects meta-analysis. 3

Table S2 Association between same-day maximum temperature and homicide by province........ 5

Table S3 Sensitivity analyses 6

Table S4 Association by age and sex between daily mean temperature and homicide 7

Table S5 Association by age and sex between daily minimum temperature and homicide 8

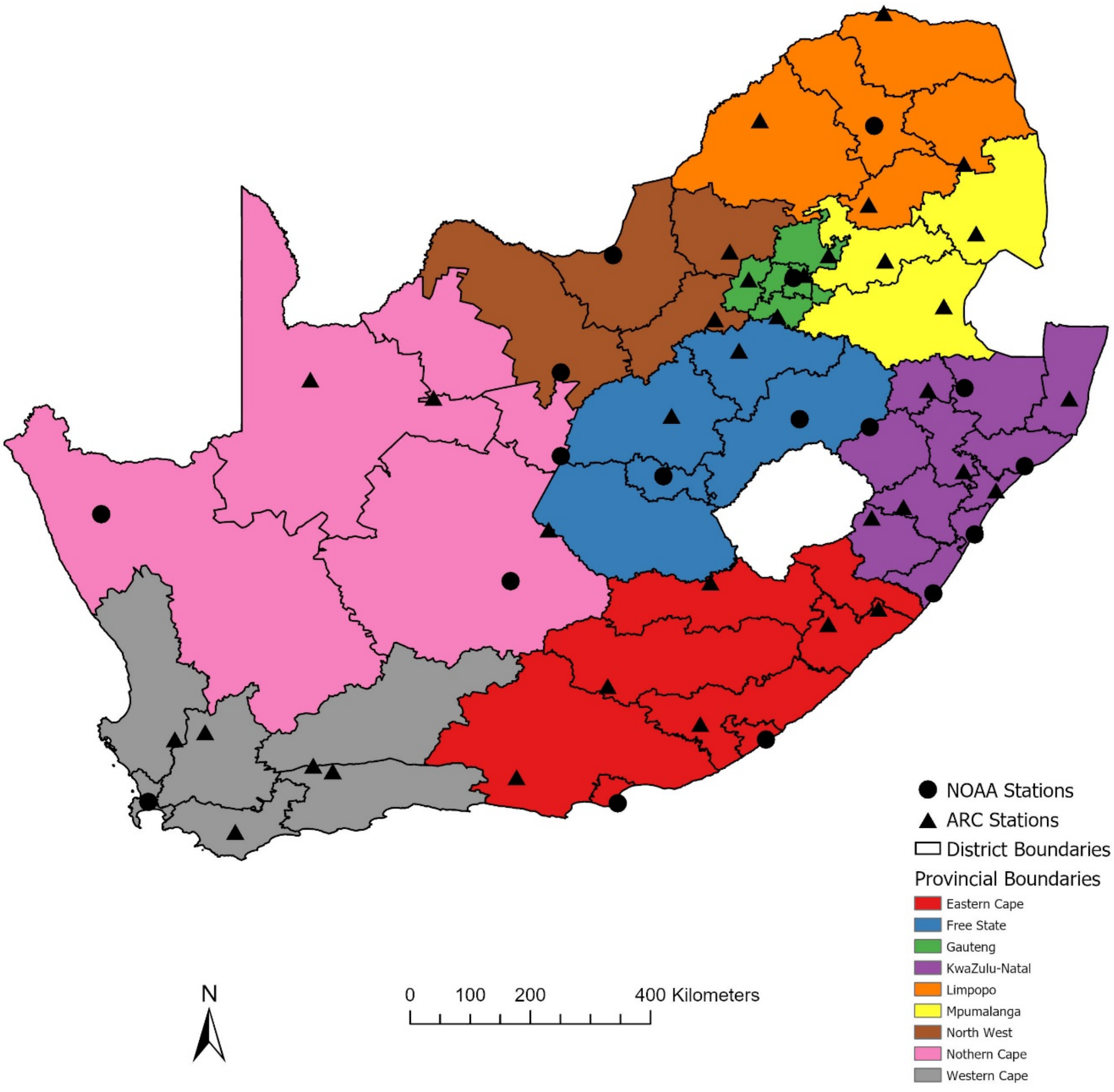

Figure S1. Provincial and district boundaries of South Africa, location of the temperature monitoring stations, and source of the data. ARC = Agricultural Research Council (South Africa), NOAA = National Oceanographic and Atmospheric Administration (USA). District boundaries are for 2011.

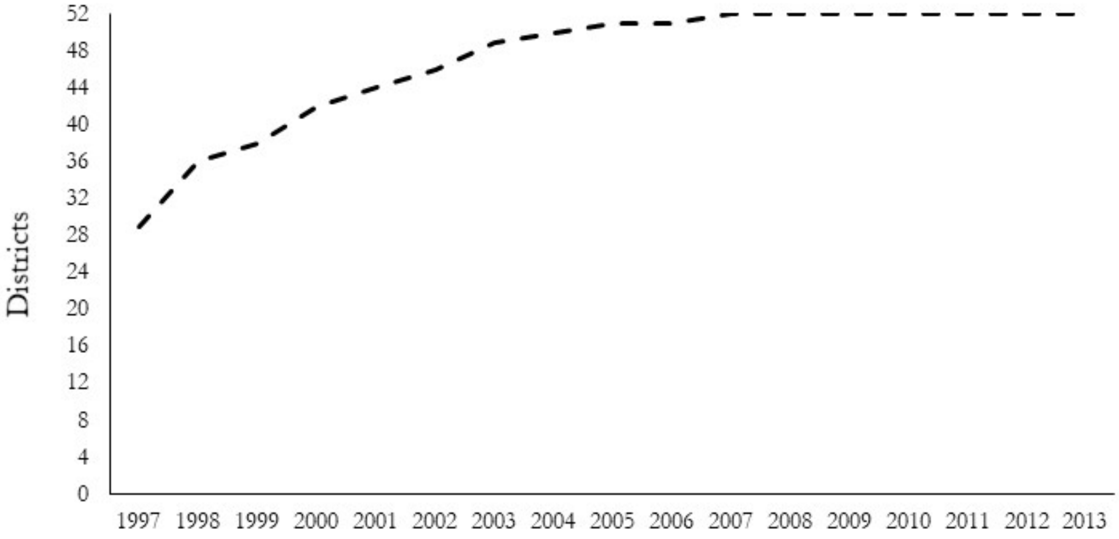

Figure S2. Number of districts (n=52) with temperature data over the study period.

Table S1

Associations between same-day maximum temperature and homicide by district, tests for interaction and heterogeneity, and overall country-level association estimated by random-effects meta-analysis. Odds ratios are reported per °C increase in temperature. Numbers in parentheses are 95% confidence intervals.

District	Province[a]	Definite homicides[b]	Total homicides[c]
Alfred Nzo	EC	1.014 (1.000, 1.027)	1.010 (1.000, 1.020)
Amajuba	KZN	1.039 (1.011, 1.066)	1.011 (0.993, 1.029)
Amathole	EC	1.010 (1.001, 1.018)	1.013 (1.008, 1.019)
Bojanala	NW	1.021 (1.001, 1.041)	1.010 (0.999, 1.022)
Buffalo City	EC	0.994 (0.978, 1.009)	0.995 (0.984, 1.005)
Cacadu	EC	0.999 (0.986, 1.012)	1.003 (0.994, 1.013)
Cape Winelands	WC	1.011 (0.999, 1.023)	1.017 (1.009, 1.024)
Capricorn	LIM	1.010 (0.982, 1.037)	1.016 (1.005, 1.027)
Central Karoo	WC	1.017 (0.988, 1.046)	1.006 (0.984, 1.030)
Chris Hani	EC	1.019 (1.006, 1.031)	1.018 (1.010, 1.027)
City of Cape Town	WC	1.018 (1.011, 1.024)	1.011 (1.007, 1.015)
City of Johannesburg	GT	1.045 (1.026, 1.064)	1.012 (1.007, 1.017)
City of Tshwane	GT	1.032 (1.008, 1.055)	1.012 (1.003, 1.022)
Dr Kenneth Kaunda	NW	1.029 (1.003, 1.055)	1.027 (1.015, 1.040)
Dr Ruth Segomotsi	NW	1.046 (1.002, 1.090)	1.023 (1.005, 1.043)
Eden	WC	1.004 (0.991, 1.017)	1.004 (0.996, 1.013)
Ehlanzeni	MP	1.014 (0.993, 1.035)	1.013 (1.000, 1.026)
Ekurhuleni	GT	1.031 (1.014, 1.049)	1.014 (1.006, 1.021)
eThekwini	KZN	1.010 (0.994, 1.025)	1.013 (1.007, 1.018)
Fezile Dabi	FS	1.034 (1.006, 1.061)	1.022 (1.001, 1.042)
Frances Baard	NC	1.034 (1.012, 1.056)	1.020 (1.004, 1.037)
Gert Sibande	MP	1.021 (1.000, 1.041)	1.010 (0.999, 1.021)
Greater Sekhukhune	LIM	0.989 (0.945, 1.033)	1.014 (0.995, 1.033)
iLembe	KZN	1.016 (0.984, 1.048)	1.020 (1.003, 1.036)
Joe Gqabi	EC	1.011 (0.983, 1.039)	1.010 (0.989, 1.032)
John Taolo Gaetsewe	NC	0.988 (0.939, 1.037)	0.986 (0.947, 1.026)
Lejweleputswa	FS	1.028 (1.010, 1.047)	1.009 (0.994, 1.023)
Mangaung	FS	1.044 (1.026, 1.062)	1.022 (1.011, 1.033)
Mopani	LIM	1.025 (1.001, 1.050)	1.004 (0.990, 1.018)
Namakwa	NC	1.024 (0.992, 1.056)	1.018 (0.993, 1.045)
Nelson Mandela Bay	EC	1.017 (1.004, 1.029)	1.014 (1.007, 1.021)
Ngaka Modiri Molema	NW	1.009 (0.979, 1.038)	1.005 (0.987, 1.023)
Nkangala	MP	1.014 (0.985, 1.043)	1.016 (1.000, 1.032)
OR Tambo	EC	1.006 (0.996, 1.016)	1.005 (1.000, 1.010)
Overberg	WC	0.994 (0.960, 1.028)	0.993 (0.968, 1.018)
Pixley ka Seme	NC	1.022 (0.997, 1.047)	1.026 (1.006, 1.047)
Sedibeng	GT	1.013 (0.989, 1.037)	1.018 (1.004, 1.032)
Sisonke	KZN	1.016 (0.998, 1.034)	1.015 (1.003, 1.027)
Siyanda	NC	1.007 (0.991, 1.022)	1.008 (0.994, 1.022)

Thabo Mofutsanyane	FS	1.017 (0.992, 1.042)	1.015 (0.998, 1.031)
Ugu	KZN	1.012 (0.987, 1.037)	1.021 (1.008, 1.035)
uMgungundlovu	KZN	1.010 (0.997, 1.023)	1.014 (1.008, 1.020)
uMkhanyakude	KZN	1.016 (0.985, 1.048)	1.014 (0.997, 1.031)
uMzinyathi	KZN	1.017 (0.997, 1.038)	1.012 (1.003, 1.020)
uThukela	KZN	1.052 (1.017, 1.087)	1.030 (1.013, 1.047)
uThungulu	KZN	1.014 (1.001, 1.027)	1.013 (1.005, 1.021)
Vhembe	LIM	1.021 (0.989, 1.053)	1.001 (0.982, 1.021)
Waterberg	LIM	0.990 (0.955, 1.025)	1.004 (0.986, 1.024)
West Coast	WC	1.014 (0.991, 1.037)	1.013 (1.003, 1.023)
West Rand	GT	1.043 (1.019, 1.067)	1.017 (1.006, 1.029)
Xhariep	FS	0.998 (0.971, 1.024)	0.998 (0.982, 1.014)
Zululand	KZN	1.025 (1.001, 1.050)	1.017 (1.002, 1.032)
Test for interaction (Cochrane Q)		p=0.008	p=0.094
Test for heterogeneity (I^2)		35.11 (8.76, 53.85)	21.17 (0.00, 44.55)
**Meta-analysis**		1.016 (1.013, 1.019)	1.012 (1.011, 1.014)

[a] EC = Eastern Cape, FS = Free State, GT = Gauteng, KZN = KwaZulu-Natal, LIM = Limpopo, MP = Mpumalanga, NC = Northern Cape, NW = Northwest, WC = Western Cape.
[b] Definite homicides, ICD-10: X85-Y09.
[c] Includes definite homicides and probable homicides (ICD-10: W25-W26, W32-W34, W50, Y22-Y24, Y28-Y29).

Table S2

Associations between same-day maximum temperature and homicide by province. Odds ratios are reported per °C increase in temperature. Numbers in parentheses are 95% confidence intervals.

District	Definite homicides[a]	Total homicides[b]
Eastern Cape	1.009 (1.005, 1.013)	1.009 (1.007, 1.012)
Free State	1.028 (1.018, 1.038)	1.014 (1.007, 1.021)
Gauteng	1.034 (1.024, 1.044)	1.013 (1.010, 1.017)
KwaZulu-Natal	1.016 (1.010, 1.022)	1.014 (1.011, 1.017)
Limpopo	1.011 (0.998, 1.025)	1.010 (1.003, 1.016)
Mpumalanga	1.017 (1.004, 1.030)	1.012 (1.005, 1.020)
Northern Cape	1.017 (1.006, 1.027)	1.015 (1.006, 1.023)
North West	1.023 (1.010, 1.037)	1.017 (1.010, 1.024)
Western Cape	1.014 (1.009, 1.019)	1.011 (1.008, 1.014)

[a] Definite homicides, ICD-10: X85-Y09.

[b] Includes definite homicides and probable homicides (ICD-10: W25-W26, W32-W34, W50, Y22-Y24, Y28-Y29).

Table S3

Sensitivity analyses: Associations between same day maximum temperature and total homicides. Each test is independent of the other tests. Numbers in parentheses are 95% confidence intervals.

**Model**	**Odds ratio**
Main model[a]	1.012 (1.011, 1.013)
Plus $PM_{2.5}$[b]	1.010 (1.003, 1.017)
Plus ozone[c]	1.011 (1.005, 1.016)
Excluding holidays	1.012 (1.011, 1.013)
Excluding 1997-2001	1.012 (1.011, 1.014)

[a] As in Table 2 of the main text.

[b] 24-hour average. Data was available for six districts.

[c] Maximum daily 8-hr running average. Data was available for five districts.

Table S4

Association by age and sex between daily mean temperature and homicide, with odds ratios reported per 1° C increase in temperature. Numbers in parentheses are 95% confidence intervals.

	Deaths included		Odds ratio	
	**Definite[b]**	**Total[c]**	**Definite[b]**	**Total[c]**
Male				
Lag 0[d]	53,625	186,282	1.021 (1.018, 1.025)	1.018 (1.016, 1.020)
Lag 1[e]	53,682	186,400	1.015 (1.012, 1.019)	1.012 (1.009, 1.014)
Lag 0-1[f]	53,277	184,922	1.023 (1.019, 1.027)	1.018 (1.016, 1.021)
Female				
Lag 0[d]	8,719	33,641	1.016 (1.007, 1.025)	1.012 (1.007, 1.017)
Lag 1[e]	8,722	33,678	1.016 (1.007, 1.025)	1.009 (1.005, 1.014)
Lag 0-1[f]	8,668	33,396	1.020 (1.010, 1.030)	1.014 (1.008, 1.019)
0-17 years				
Lag 0[d]	4,067	14,150	1.014 (1.000, 1.027)	1.011 (1.004, 1.019)
Lag 1[e]	4,071	14,170	1.016 (1.003, 1.030)	1.011 (1.003, 1.018)
Lag 0-1[f]	4,039	14,051	1.019 (1.004, 1.034)	1.013 (1.005, 1.022)
18-44 years				
Lag 0[d]	47,617	160,465	1.020 (1.016, 1.024)	1.018 (1.016, 1.020)
Lag 1[e]	47,657	160,548	1.014 (1.010, 1.018)	1.011 (1.009, 1.013)
Lag 0-1[f]	47,308	159,271	1.021 (1.017, 1.026)	1.018 (1.015, 1.020)
45+ years				
Lag 0[d]	10,313	44,228	1.026 (1.018, 1.034)	1.015 (1.011, 1.019)
Lag 1[e]	10,332	44,278	1.023 (1.015, 1.032)	1.013 (1.009, 1.017)
Lag 0-1[f]	10,254	43,924	1.031 (1.021, 1.040)	1.018 (1.013, 1.022)

a Here the mean is defined as the average between the maximum and the minimum.

[b] Definite homicides, ICD-10: X85-Y09.

[c] Includes definite homicides and probable homicides (ICD-10: W25-W26, W32-W34, W50, Y22-Y24, Y28-Y29).

[d] Same day's temperature.

[e] Previous day's temperature.

[f] Moving average of same and previous day's temperature.

Table S5

Association by age and sex between daily minimum temperature and homicide, with odds ratios reported per 1° C increase in temperature. Numbers in parentheses are 95% confidence intervals.

	**Deaths included**		**Odds ratio**	
	**Definite**[a]	**Total**[b]	**Definite**[a]	**Total**[b]
Male				
Lag 0[c]	53,827	187,001	1.010 (1.007, 1.014)	1.010 (1.008, 1.011)
Lag 1[d]	53,877	187,129	1.007 (1.004, 1.010)	1.006 (1.004, 1.008)
Lag 0-1[e]	53,532	185,822	1.012 (1.008, 1.015)	1.010 (1.008, 1.012)
Female				
Lag 0[c]	8,763	33,786	1.008 (0.999, 1.016)	1.005 (1.001, 1.010)
Lag 1[d]	8,755	33,814	1.006 (0.998, 1.015)	1.004 (0.999, 1.008)
Lag 0-1[e]	8,712	33,570	1.009 (1.000, 1.019)	1.006 (1.000, 1.011)
0-17 years				
Lag 0[c]	4,082	14,206	0.999 (0.997, 1.011)	1.002 (0.995, 1.009)
Lag 1[d]	4,088	14,225	1.005 (0.993, 1.017)	1.002 (0.995, 1.008)
Lag 0-1[e]	4,058	14,116	1.003 (0.989, 1.017)	1.002 (0.995, 1.010)
18-44 years				
Lag 0[c]	47,801	161,106	1.009 (1.006, 1.013)	1.010 (1.008, 1.012)
Lag 1[d]	47,825	161,189	1.006 (1.003, 1.010)	1.006 (1.004, 1.008)
Lag 0-1[e]	47,532	160,080	1.010 (1.006, 1.015)	1.010 (1.008, 1.013)
45+ years				
Lag 0[c]	10,363	44,399	1.018 (1.010, 1.025)	1.007 (1.004, 1.011)
Lag 1[d]	10,374	44,449	1.013 (1.005, 1.020)	1.006 (1.002, 1.010)
Lag 0-1[e]	10,311	44,128	1.020 (1.011, 1.029)	1.009 (1.005, 1.014)

[a] Definite homicides, ICD-10: X85-Y09.
[b] Includes definite homicides and probable homicides (ICD-10: W25-W26, W32-W34, W50, Y22-Y24, Y28-Y29).
[c] Same day's temperature.
[d] Previous day's temperature.
[e] Moving average of same and previous day's temperature.
